# Semiquantitative analysis of cerebral [^18^F]FDG-PET uptake in pediatric patients

**DOI:** 10.1007/s00247-023-05794-4

**Published:** 2023-11-01

**Authors:** Álvaro Cruz-Cortes, Arturo Avendaño-Estrada, Sarael Alcauter, Juan Carlos Núñez-Enríquez, Belen Rivera-Bravo, Miguel Ángel Olarte-Casas, Miguel Ángel Ávila-Rodríguez

**Affiliations:** 1 Unidad de Radiofarmacia-Ciclotrón, División de Investigación, Facultad de Medicina, Universidad Nacional Autónoma de México, Ciudad de Mexico, Mexico; 2 Instituto de Neurobiología, Universidad Nacional Autónoma de México, Querétaro City, Mexico; 3 Unidad de Investigación Médica en Epidemiología Clínica, UMAE Hospital de Pediatría, Centro Médico Nacional Siglo XXI, Instituto Mexicano del Seguro Social, Ciudad de Mexico, Mexico; 4 División de Investigación Facultad de Medicina Universidad Nacional Autónoma de México, Unidad PET/CT, Ciudad de Mexico, Mexico

**Keywords:** [^18^F]FDG-PET, Brain metabolism, SUV, Development, Neuroimaging, Children brain

## Abstract

**Background:**

Glycolytic metabolism in the brain of pediatric patients, imaged with [^18^F]  fluorodeoxyglucose-positron emission tomography (FDG-PET) is incompletely characterized.

**Objective:**

The purpose of the current study was to characterize [^18^F]FDG-PET brain uptake in a large sample of pediatric patients with non-central nervous system diseases as an alternative to healthy subjects to evaluate changes at different pediatric ages.

**Materials and Methods:**

Seven hundred ninety-five [^18^F]FDG-PET examinations from children < 18 years of age without central nervous system diseases were included. Each brain image was spatially normalized, and the standardized uptake value (SUV) was obtained. The SUV and the SUV relative to different pseudo-references were explored as a function of age.

**Results:**

At all evaluated ages, the occipital lobe showed the highest [^18^F]FDG uptake (0.27 ± 0.04 SUV/year), while the parietal lobe and brainstem had the lowest uptake (0.17 ± 0.02 SUV/year, for both regions). An increase [^18^F]FDG uptake was found for all brain regions until 12 years old, while no significant uptake differences were found between ages 13 (SUV = 5.39) to 17 years old (SUV = 5.52) (*P* < 0.0001 for the whole brain). A sex dependence was found in the *SUVmean* for the whole brain during adolescence (SUV 5.04–5.25 for males, 5.68–5.74 for females, *P* = 0.0264). Asymmetries in [^18^F]FDG uptake were found in the temporal and central regions during infancy.

**Conclusions:**

Brain glycolytic metabolism of [^18^F]FDG, measured through the *SUVmean*, increased with age until early adolescence (< 13 years old), showing differences across brain regions. Age, sex, and brain region influence [^18^F]FDG uptake, with significant hemispheric asymmetries for temporal and central regions.

**Graphical Abstract:**

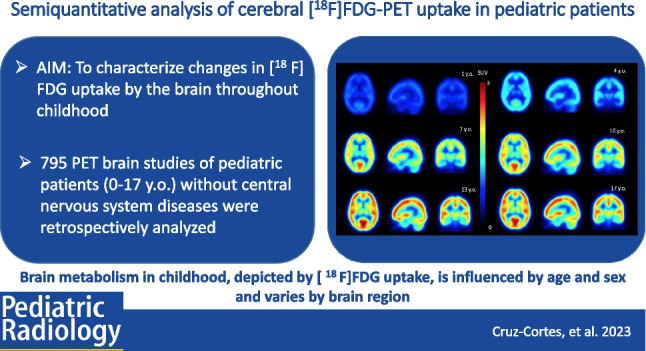

## Introduction

Pediatric neurodevelopment is a dynamic and complex process driven by multiple physiological changes, including myelinization, synaptic genesis, pruning, hormonal, and neurotransmission changes which are expected to impact glycolytic metabolism. Neuroimaging is an excellent tool for evaluating these changes during brain development in a noninvasive way [1–3].

Knowledge of the normal patterns of brain metabolism during childhood is essential to understand brain development and for identifying pathological alterations of metabolic and morphologic brain changes by imaging [4–11]. For example, imaging has demonstrated diagnostic utility in diseases such as epilepsy, autism, brain tumors, and psychiatric disorders [8, 12–17]. Despite the usefulness of neuroimaging in pediatric patients, there is a paucity of information about normal and abnormal brain structure and function.

The most used radiotracer in positron emission tomography (PET) imaging worldwide to assess, indirectly, glycolytic metabolism is [^18^F]fluoro-2-deoxy-D-glucose ([^18^F]FDG-PET), which is most commonly used for staging and monitoring response to treatment of neoplasms. In recent years, the use of quantitative methods and automatic segmentation has led to a growing number of applications of [^18^F]FDG-PET for brain imaging, including the study of resting-state brain connectivity [18–21]. Furthermore, the diagnosis of psychiatric and neurological disorders has benefited from using [^18^F]FDG-PET imaging in children [22–25].

Quantitative or semiquantitative analysis of pediatric brain [^18^F]FDG-PET images could help discriminate abnormal from normal uptake. Such analyses are complicated by slight normal variations in locoregional uptake which may be misinterpreted as a pathological condition. Therefore, normal uptake references are necessary for pediatric brain [^18^F]FDG-PET uptake. However, the study of normal brain metabolism in childhood is complex for two main reasons: (1) from an ethical standpoint, healthy pediatric subjects cannot be exposed to ionizing radiation and (2) a considerably large number of subjects is necessary to optimally characterize the age-related changes in brain metabolism [26, 27].

Efforts to characterize brain changes during childhood have been performed using absolute and relative values of regional brain uptake, using a variety of developmental models, including linear [28–30], polynomial, and transcendental functions [26, 31]. It has been reported that [^18^F]FDG uptake in the cerebral cortex tends to be higher at an early age due to neuronal proliferation and overpopulation, followed by synaptic stability and a decrease during adolescence characterized by synaptic pruning [32, 33]. Likewise, pediatric metabolic brain connectivity [34] has been studied in order to evaluate integration and functional segregation trajectories. Studies on cortical anatomical development [35] and functional networks [36] in childhood have also been performed. These previous studies have reported an increase in [^18^F]FDG-PET uptake with age. However, most studies have had limited sample sizes or included limited age ranges.

The purpose of the current study was to characterize [^18^F]FDG-PET brain uptake in a large sample of pediatric patients with non-central nervous system diseases as an alternative to healthy subjects to evaluate changes at different pediatric ages: infancy (0–4 years old), childhood (5–11 years old), and adolescence (12–19 years old) [37, 38]. Semiquantitative measurement with the standardized uptake value (SUV) was used to analyze the metabolism at each age [39].

## Methods

This research project retrospectively included data acquired for clinical indications. Ethics approval was obtained from the Ethics in Research Committee of the Research Division, School of Medicine, Universidad Nacional Autónoma de México (UNAM) under the reference number SR1132014. This study was performed in accordance with the ethical standards of the 1964 Declaration of Helsinki and its later amendments. In addition, written informed consent was obtained from each child’s parents or guardians, permitting the use of data for both clinical and research purposes.

### Image acquisition

Whole-body [^18^F]FDG-PET/CT scans were acquired on a Siemens Biograph Truepoint 64 (Siemens Medical Systems, USA). Verification of the cross-calibration between the PET scanner and the dose calibrator was periodically performed by using a uniform phantom filled by a ^18^F solution as part of the quality control of the scanner. Each subject received a weight-adapted dose of 6.95 ± 1.87 MBq/kg of [^18^F]FDG intravenously after fasting for at least 6 h; patients < 2 years old fasted for approximately 4 h. Patients that required sedation for clinical imaging had nothing to eat or drink after midnight before the examination. In all cases, the patients fasted during the 1-h [^18^F]FDG uptake period. When required, anesthesia was administered during the scan acquisition, after the biodistribution period of the radiopharmaceutical.

Once the radiopharmaceutical was administered, patients remained in the preparation room and rested for approximately 70 min (range 60 to 79 min). A transmission CT scan (35 mAs, 120 kVs, slice thickness of 3 mm) was used for attenuation correction, followed by a PET emission scan for 2 min/bed position from the skull to the proximal third of the thigh. PET images were reconstructed with an ordered subset expectation maximization algorithm 2D (3–4 iterations, 15 subsets, Gaussian filter of 8–10 mm). The voxel size of the image was 4.07 × 4.07 × 3 mm^3^.

### Patient selection

The imaging database of the PET/CT Unit of the School of Medicine at the UNAM, Research Division, was used as the source of analyzed data in the present research with whole-body [^18^F]FDG-PET/CT studies performed on pediatric patients younger than 18 years of age between January 2010 and February 2019 included. A senior M.D. specialist (NE, 18 years experience) reviewed the clinical information of each patient in order to select the sample of subjects for the present analysis.

The following cases were excluded from the study: patients with a history or presence of a neurological disease, central nervous system (CNS) involvement by neoplasms or infections, surgery or biopsy at brain level, diabetes, vasculitis, and prior or current treatment with chemotherapy or intrathecal radiotherapy. Additionally, examinations were excluded for incomplete clinical data, images that did not cover the entire brain, presence of artifacts, uptake times > 80 min, follow-up studies of the same patient, images with SUVmean outlier values based on a robust regression, and outlier removal test [40] with a Q = 5%.

### Image processing

Brain images for the selected patients were extracted from the whole-body PET/CT images using the PMOD v.3.806 software (PMOD Technologies LLC). Once the brain images were obtained, spatial normalization and co-registration procedures were performed through SPM12 v. 9.11 (Institute of Neurology, London, UK) [41]. Normalized images were segmented using the Hammers N30R83 atlas implemented in PMOD [42] to obtain the 3D volume of interest (VOI) of 83 cortical and subcortical brain structures and were scaled to their SUV using the injected dose and the weight of each patient. The average SUV (*SUV*_*mean*_) was obtained in each structure for all subjects. In addition, an average FDG uptake brain map (in terms of *SUVmean*) was created for each age group through all the subject’s data (freely available).

### Statistical analysis

A linear regression analysis and standard deviation of the $SU{V}_{mean}$ values by age was performed to investigate the relationship of the [^18^F]FDG uptake across the pediatric age ranges. To evaluate the effect of age on [^18^F]FDG uptake in each region, an ANOVA test was performed with multiple Tukey–Kramer post hoc comparisons between $SU{V}_{mean}$ values and age groups. Multiple linear and non-linear models were proposed for each region to model the age effect on regional glycolytic brain metabolism. The models considered were linear, quadratic, cubic, exponential ($y={y}_{0}{e}^{K*age}$), and power law ($y=A*{age}^{B}+C{*age}^{D}$) at 95% prediction interval. The optimal model for each brain region was determined through the lowest Akaike information criterion (AIC), root mean square error (RMSE) value, and coefficient of determination (${R}^{2}$) closest to unity.

The sex effect on the [^18^F]FDG brain uptake in each region was evaluated using a two-way ANOVA test and post hoc multiple comparisons (Bonferroni), comparing the $SU{V}_{mean}$ of males and females in each age group.

In addition, for each age group, the regional $SU{V}_{mean}$ normalized to pseudo-references was evaluated using previously reported regions [26, 28, 31]: cerebellum, brainstem, and the whole brain. Relative SUV ($SU{V}_{rel}$) was defined as the ratio of the *SUVmean* to the $SU{V}_{mean}$ of the pseudo-reference.

The effect of laterality on the [^18^F]FDG uptake was evaluated from the values of the 37 paired brain structures in each age group, and the structures whose absolute percentage difference of *SUV*_*mean*_ between left- and right-sided was more than 5% ($\left|\Delta {SUV}_{L,R}\right|$> 5%) were evaluated. First, a normality test (Kolmogorov Smirnov, *n* > 50 and Shapiro Wilk, *n* < 50) was performed for this evaluation. Subsequently, once the type of distribution of the data was known, the corresponding statistical tests were performed (paired *t*-test or Wilcoxon’s test).

In order to avoid systematic bias in the [^18^F]FDG uptake semi-quantification due to the large number of patients with Hodgkin’s lymphoma in this sample, an unpaired *t*-test with Welch’s correction was performed to compare the mean regional $SU{V}_{mean}$ values of this group of patients to other included patients.

All statistical analyses were carried out using GraphPad Prism v. 8.0.1 and R Studio v.1.4.1717 (R-Project.org) with *p* < 0.05 (*α* = 5%) considered significant for all inference testing.

## Results

**Fig. 1:**
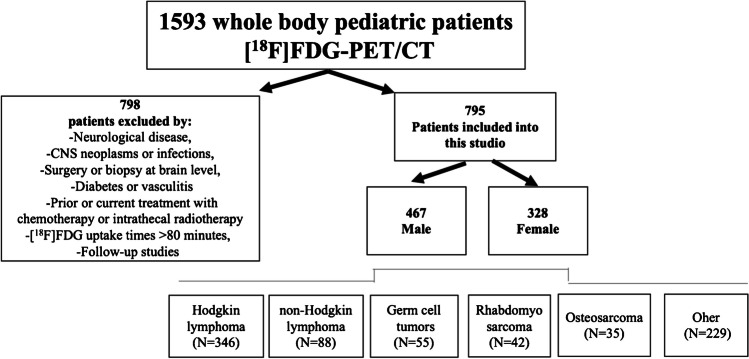
Population sample flow diagram

**Fig. 2:**
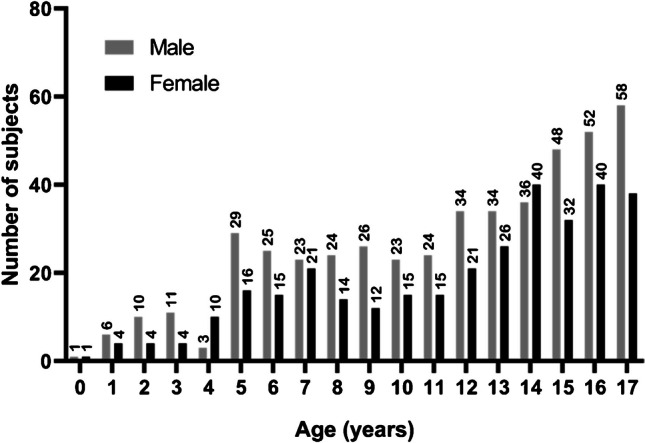
Age and sex distribution of the included patients

A total of 1593 whole-body [^18^F]FDG-PET/CT studies were performed during the study period. Based on exclusion criteria (Fig. 1), imaging data from 795 patients (328 females and 467 males) were included in the analysis, with a median age of 13 years. The most common diagnoses of the study sample were Hodgkin lymphoma (346/795), non-Hodgkin lymphoma (88/795), testicular/ovarian germ cell tumors (55/795), rhabdomyosarcoma (42/795), and osteosarcoma (35/795). Figure 2 shows the age and sex distribution of patients.

### Average [^18^F]FDG brain uptake for evaluated ages

**Fig. 3:**
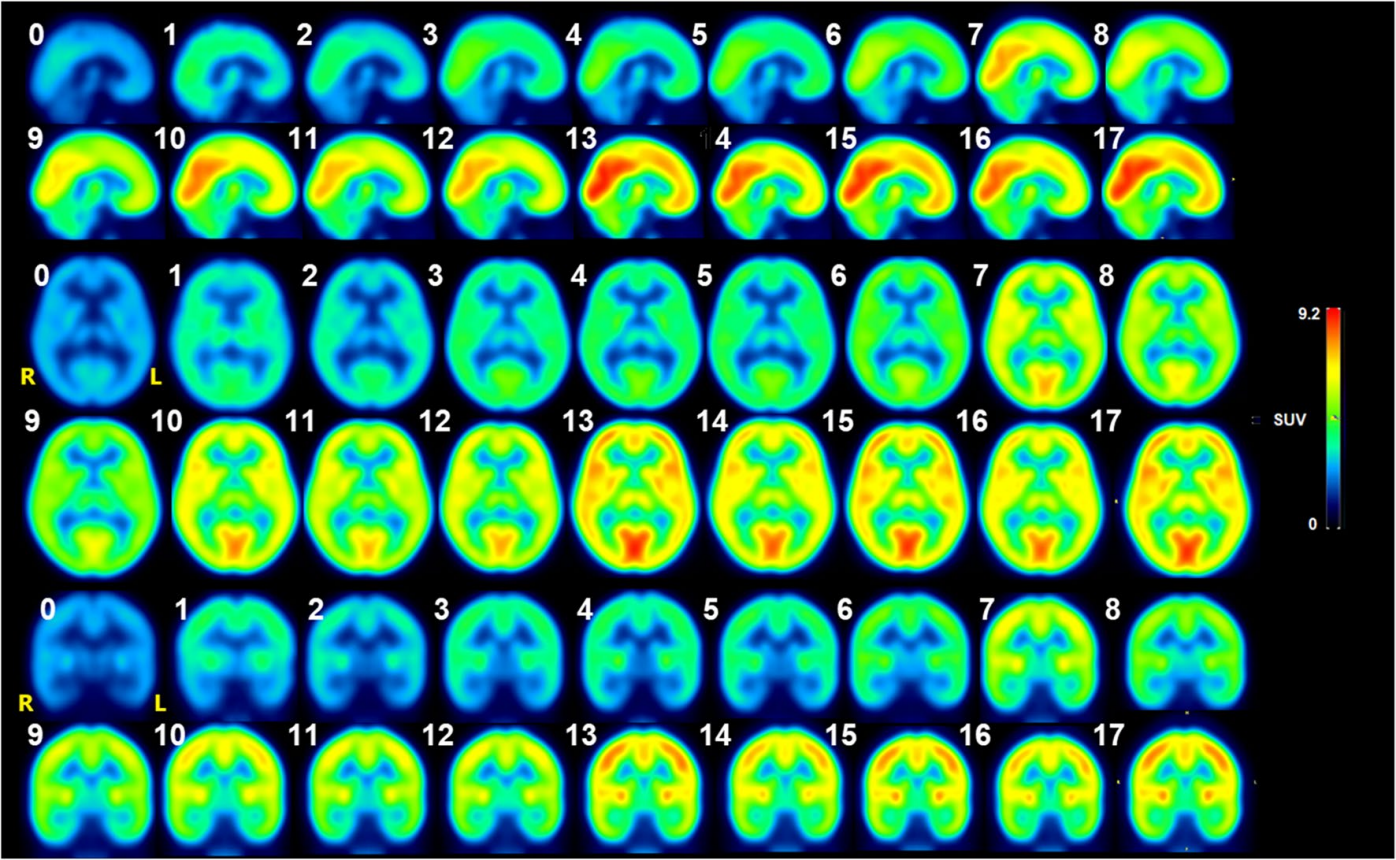
Average [^18^F]FDG-PET brain uptake image (in terms of SUV) displayed as sagittal, transverse, and coronal brain sections. Numbers indicate years of age

Figure 3 shows images of the average [^18^F]FDG brain uptake for all the evaluated ages. Glycolytic metabolism is observed to increase with age [28]. On average across all age groups, the occipital lobe showed 24% higher uptake than the whole brain average, followed by the cingulate gyrus (10%) and parietal lobe structures (6%). The uptake in the brainstem was 29% lower than the average whole brain, followed by the temporal lobe (18%) and the cerebellum (8%). The structures with more variability were the cingulate gyrus (CV = 31.81%), central structures (CV = 31.3%), and occipital lobe (CV = 30.9%). There was no significant difference in regional ${\varvec{S}}{\varvec{U}}{{\varvec{V}}}_{{\varvec{m}}{\varvec{e}}{\varvec{a}}{\varvec{n}}}$ (*p* > 0.9999) for any of the studied regions between subgroups of patients with Hodgkin’s lymphoma and without Hodgkin’s lymphoma.

### SUV_mean_ changes associated with age and sex

**Fig. 4:**
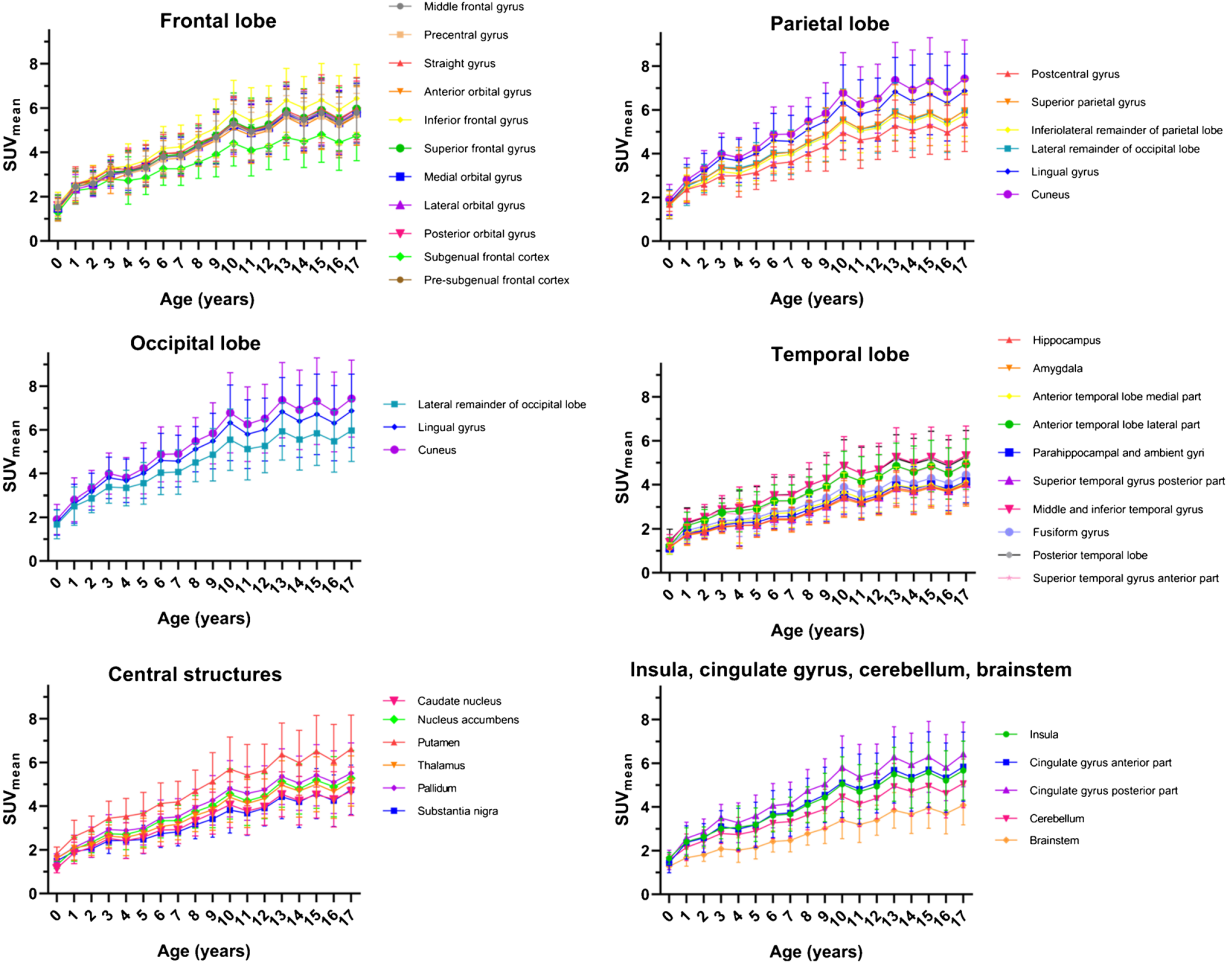
Trends in the average [^18^F]-FDG uptake (${\mathrm{SUV}}_{\mathrm{mean}}$) in the different brain regions by age

The semiquantitative data obtained from the different brain regions and ages are shown in Fig. 4. As age increases, there is an increase in the $SU{V}_{mean}$ with a monotonic trend in all brain structures. In addition, a continuous increase in the [^18^F]FDG brain uptake is observed between ages 0 and 12, which corresponds to infant, preschool, and school-age children, while in 13-year-old children and up, uptake is relatively constant in different brain regions.

Based on linear regression analysis, the regions with the highest and the lowest [^18^F]FDG uptake are the occipital lobe (0.27 ± 0.04 SUV/year, ${R}^{2}$=0.9231) and the parietal lobe (0.17 ± 0.02 SUV/year, ${R}^{2}$=0.9241), respectively. Other structures with relatively high [^18^F]FDG uptake were the cuneus (0.31 ± 0.05 SUV/year, ${R}^{2}$=0.9290), followed by the lingual gyrus (0.28 ± 0.04 SUV/year, ${R}^{2}$=0.9207) and inferior frontal gyrus (0.27 ± 0.04 SUV/year, ${R}^{2}$=0.9352), while the amygdala (0.15 ± 0.02 SUV/year, ${R}^{2}$=0.9627), hippocampus (0.16 ± 0.02 SUV/year, ${R}^{2}$=0.959), and brainstem (0.16 ± 0.02 SUV/year, ${R}^{2}$=0.9665) had a relatively lower [^18^F]FDG uptake. On average, SUV of the whole brain changed at the rate of 0.22 ± 0.03 SUV/year with ${R}^{2}$=0.9396. Table 1 shows normal values of regional FDG uptake by semiquantitative measurement.

**Table 1:** $SU{V}_{mean}$ values (95% CI) for different brain structures for each age group

Age (years)	Frontal lobe	Temporal lobe	Parietal lobe	Occipital lobe	Central structures	Insula	Cerebellum	Whole brain	Cingulate gyrus	Brainstem
0	1.51 (1.35; 1.67)	1.22 (1.06; 1.38)	1.65 (1.61; 1.69)	1.78 (1.62; 1.94)	1.50 (1.17; 1.83)	1.65 (1.27; 2.03)	1.56 (1.20; 1.92)	1.52 (1.28; 1.76)	1.51 (0.88; 2.14)	1.28 (1.27; 1.29)
1	2.44 (2.38; 2.50)	1.97 (1.83; 2.11)	2.46 (2.39; 2.53)	2.64 (2.55; 2.73)	2.10 (1.94; 2.26)	2.40 (2.00; 2.80)	2.15 (1.77; 2.53)	2.28 (2.10; 2.46)	2.47 (2.01; 2.93)	1.67 (1.43; 1.91)
2	2.65 (2.58; 2.72)	2.17 (2.01; 2.33)	2.71 (2.64; 2.78)	3.12 (3.00; 3.24)	2.36 (2.19; 2.53)	2.63 (2.39; 2.87)	2.44 (2.17; 2.71)	2.55 (2.37; 2.73)	2.71 (2.38; 3.04)	1.81 (1.65; 1.97)
3	3.07 (2.97; 3.17)	2.43 (2.25; 2.61)	3.17 (3.08; 3.26)	3.73 (3.57; 3.89)	2.78 (2.59; 2.97)	3.00 (2.76; 3.24)	2.78 (2.55; 3.01)	2.97 (2.74; 3.20)	3.29 (2.94; 3.64)	2.08 (1.90; 2.26)
4	3.11 (3.02; 3.20)	2.48 (2.30; 2.66)	3.12 (3.04; 3.20)	3.61 (3.48; 3.74)	2.76 (2.53; 2.99)	3.07 (2.54; 3.60)	2.73 (2.30; 3.16)	2.94 (2.71; 3.17)	3.13 (2.62; 3.64)	2.02 (1.71; 2.33)
5	3.32 (3.26; 3.38)	2.56 (2.45; 2.67)	3.35 (3.30; 3.40)	3.93 (3.83; 4.03)	2.90 (2.78; 3.02)	3.19 (2.97; 3.41)	2.90 (2.70; 3.10)	3.13 (2.98; 3.28)	3.38 (3.09; 3.67)	2.14 (1.99; 2.29)
6	3.79 (3.72; 3.86)	2.89 (2.75; 3.03)	3.79 (3.72; 3.86)	4.50 (4.37; 4.63)	3.27 (3.12; 3.42)	3.60 (3.37; 3.83)	3.27 (3.05; 3.49)	3.55 (3.37; 3.73)	3.86 (3.54; 4.18)	2.42 (2.27; 2.57)
7	3.84 (3.77; 3.91)	2.91 (2.77; 3.05)	3.88 (3.81; 3.95)	4.51 (4.39; 4.63)	3.33 (3.18; 3.48)	3.67 (3.44; 3.90)	3.33 (3.11; 3.55)	3.60 (3.43; 3.77)	3.93 (3.63; 4.23)	2.46 (2.30; 2.62)
8	4.26 (4.17; 4.35)	3.24 (3.08; 3.40)	4.32 (4.23; 4.41)	5.03 (4.87; 5.19)	3.73 (3.56; 3.90)	4.10 (3.84; 4.36)	3.63 (3.41; 3.85)	4.02 (3.81; 4.23)	4.46 (4.13; 4.79)	2.77 (2.60; 2.94)
9	4.63 (4.54; 4.72)	3.51 (3.34; 3.68)	4.65 (4.56; 4.74)	5.39 (5.23; 5.55)	4.06 (3.87; 4.25)	4.43 (4.08; 4.78)	3.90 (3.61; 4.19)	4.34 (4.12; 4.56)	4.79 (4.40; 5.18)	3.01 (2.79; 3.23)
10	5.22 (5.12; 5.32)	3.99 (3.80; 4.18)	5.32 (5.22; 5.42)	6.21 (6.01; 6.41)	4.56 (4.35; 4.77)	5.04 (4.64; 5.44)	4.48 (4.11; 4.85)	4.94 (4.68; 5.20)	5.45 (4.96; 5.94)	3.39 (3.12; 3.66)
11	4.88 (4.78; 4.98)	3.71 (3.54; 3.88)	4.92 (4.83; 5.01)	5.72 (5.54; 5.90)	4.30 (4.10; 4.50)	4.70 (4.31; 5.09)	4.14 (3.81; 4.47)	4.59 (4.36; 4.82)	5.09 (4.60; 5.58)	3.20 (2.94; 3.46)
12	5.10 (5.01; 5.19)	3.92 (3.78; 4.06)	5.09 (5.01; 5.17)	5.93 (5.76; 6.10)	4.51 (4.34; 4.68)	4.93 (4.65; 5.21)	4.4 (4.15; 4.65)	4.81 (4.61; 5.01)	5.34 (4.99; 5.69)	3.39 (3.21; 3.57)
13	5.69 (5.59; 5.79)	4.38 (4.23; 4.53)	5.63 (5.55; 5.71)	6.70 (6.52; 6.88)	5.12 (4.94; 5.30)	5.49 (5.18; 5.80)	4.94 (4.67; 5.21)	5.39 (5.18; 5.60)	5.98 (5.61; 6.35)	3.85 (3.64; 4.06)
14	5.38 (5.30; 5.46)	4.18 (4.06; 4.30)	5.37 (5.3; 5.44)	6.29 (6.14; 6.44)	4.82 (4.67; 4.97)	5.24 (4.95; 5.53)	4.70 (4.44; 4.96)	5.10 (4.93; 5.27)	5.64 (5.30; 5.98)	3.66 (3.46; 3.86)
15	5.74 (5.66; 5.82)	4.41 (4.29; 4.53)	5.64 (5.57; 5.71)	6.62 (6.46; 6.78)	5.2 (5.04; 5.36)	5.57 (5.26; 5.88)	4.96 (4.68; 5.24)	5.42 (5.25; 5.59)	6.00 (5.63; 6.37)	3.95 (3.74; 4.16)
16	5.32 (5.25; 5.39)	4.15 (4.05; 4.25)	5.26 (5.21; 5.31)	6.2 (6.06; 6.34)	4.88 (4.74; 5.02)	5.21 (4.93; 5.49)	4.63 (4.40; 4.86)	5.06 (4.91; 5.21)	5.57 (5.25; 5.89)	3.70 (3.51; 3.89)
17	5.77 (5.69; 5.85)	4.52 (4.41; 4.63)	5.71 (5.65; 5.77)	6.76 (6.61; 6.91)	5.33 (5.19; 5.47)	5.66 (5.39; 5.93)	5.08 (4.86; 5.30)	5.52 (5.36; 5.68)	6.12 (5.81; 6.43)	4.08 (3.90; 4.26)

For most brain regions, no statistically significant differences were found in [^18^F]FDG uptake by age between infant and preschool age. Similarly, in middle adolescence (13–17 years old), most regions have no significant differences from the average SUV. However, significant differences were observed when comparing the average SUV in the 0 to 12 age group to the average SUV of older subjects (> 12 years old).

**Fig. 5:**
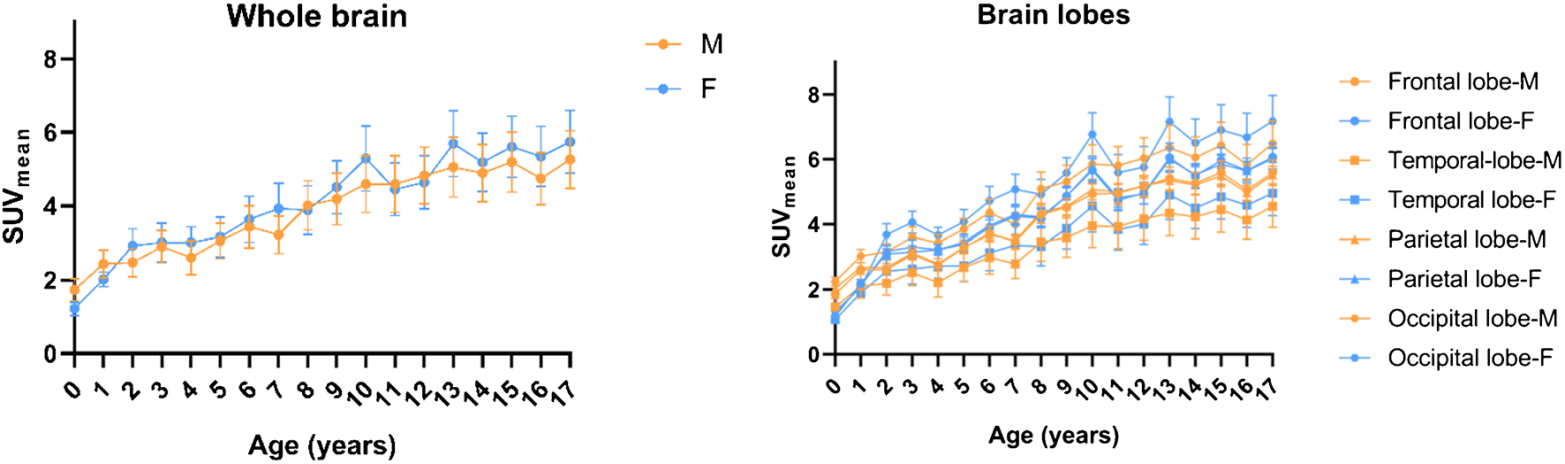
Comparison of whole brain and lobar $SU{V}_{mean}$ changes in male (M) and female (F) groups

**Fig. 6:**
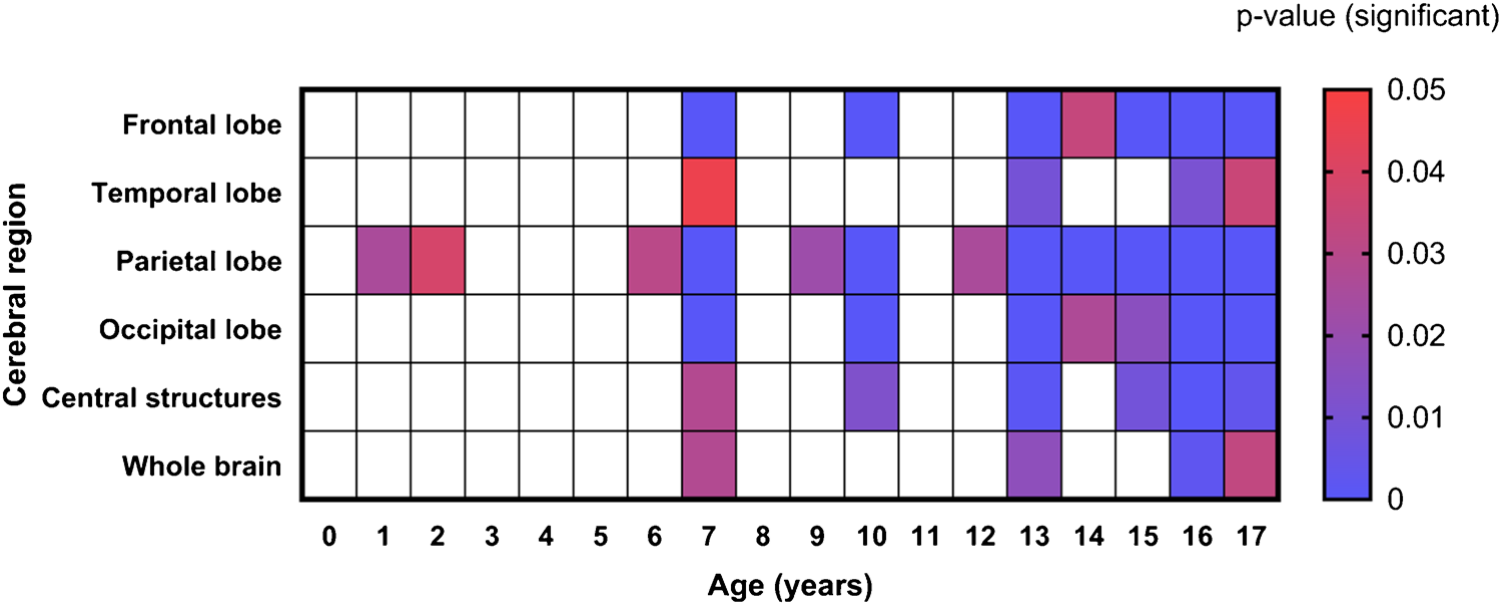
Matrix of *P*-values (scaled by color) for comparison of lobar ${\mathrm{SUV}}_{\mathrm{mean}}$ by sex (male vs. female) at each year of age

For most age groups, females had a higher average [^18^F]FDG brain uptake than males in all lobes and whole brain (Fig. 5). Figure 6 graphically depicts the significance of lobar differences in [^18^F]FDG uptake by sex according to patient age. Significant differences by sex were apparent at ages of 7, 13, 16, and 17 years old.

### Adjustment models on regional SUV_mean_ associated with age

**Fig. 7:**
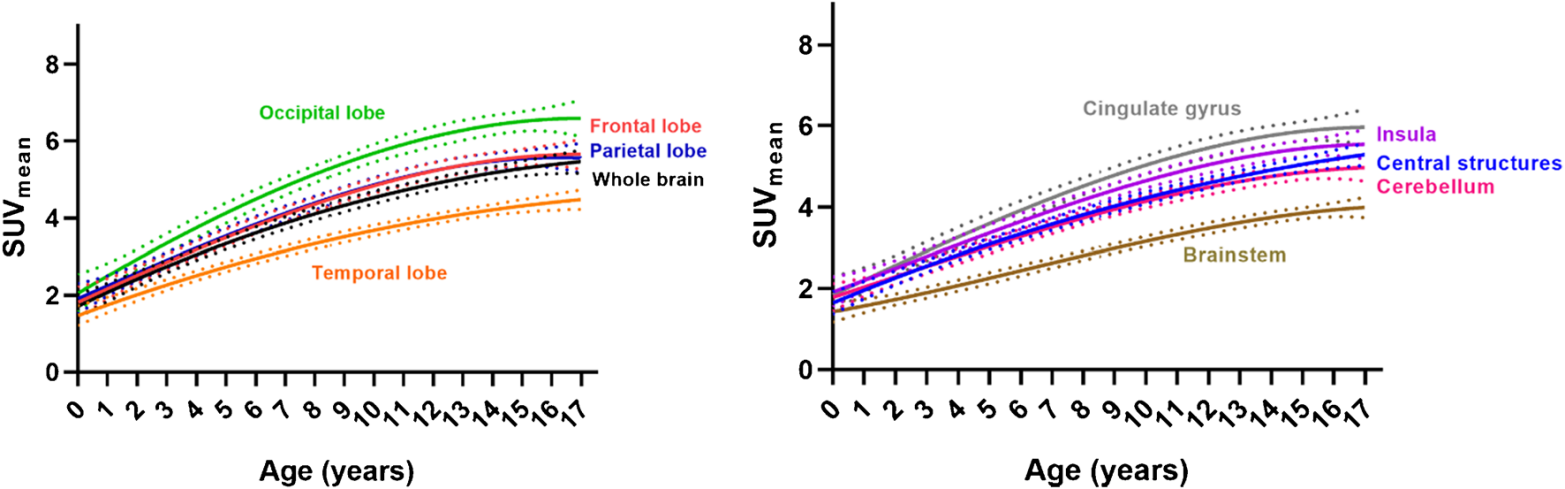
Fitting curves for $SU{V}_{mean}$ trend (solid line) by age for whole brain and lobes (left) and subcortical as well as caudal structures (right). The 95% prediction interval for each curve is represented by the dashed line bands

According to age, the optimal $SU{V}_{mean}$ models were cubic in 7 regions (frontal, parietal, occipital lobe, insula, cerebellum, cingulate gyrus, brainstem) and quadratic in 3 regions (temporal lobe, central structures, whole brain) (Fig. 7). Table 2 summarizes the fit parameters and model consideration data. A considerable increase in $SU{V}_{mean}$ is observed in the models throughout infancy to middle adolescence, after which the increase diminishes. In addition, the curve associated with the model in the occipital lobe was above all other regions, while the curve associated with the model in the brainstem was below the others. The results suggest that model curve peaks are outside the pediatric age range (< 17 years old).

**Table 2:** Optimal models data and parameters for all regions of $SU{V}_{mean}$ analysis. The quadratic and cubic models are represented by the equations ${SUV}_{mean}(age)= A+B*age+C*{age}^{2}$ and ${SUV}_{mean}(age)= A+B*age+C*{age}^{2}+D*{age}^{3}$, respectively. Each fit parameter is associated with its error fit value

Region	Frontal lobe	Temporal lobe	Parietal lobe	Occipital lobe	Central structures	Insula	Cerebellum	Whole brain	Cingulate gyrus	Brainstem
	Cubic	Quadratic	Cubic	Cubic	Quadratic	Cubic	Cubic	Quadratic	Cubic	Cubic
Adjustment parameters										
A	1.815 ± 0.395	1.471 ± 0.255	1.912 ± 0.39	2.053 ± 0.481	1.645 ± 0.272	1.913 ± 0.367	1.783 ± 0.337	1.713 ± 0.311	1.843 ± 0.452	1.427 ± 0.254
B	0.3528 ± 0.2073	0.2834 ± 0.0696	0.3319 ± 0.2048	0.4532 ± 0.2519	0.3201 ± 0.0742	0.2849 ± 0.1925	0.2493 ± 0.1771	0.369 ± 0.0396	0.3619 ± 0.2372	0.1366 ± 0.1333
C	− 0.0016 ± 0.0288	− 0.0063 ± 0.004	0.0009 ± 0.0267	− 0.0062 ± 0.035	− 0.0062 ± 0.0042	0.0032 ± 0.0203	0.0019 ± 0.0208	− 0.0087 ± 0.0072	− 0.0004 ± 0.033	0.0077 ± 0.003
D	− 0.0003 ± 0.0011		− 0.0005 ± 0.0011	− 0.0003 ± 0.0014		− 0.0004 ± 0.001	− 0.0003 ± 0.001		− 0.0004 ± 0.0013	− 0.0004 ± 0.0007
Adjustment data										
Degrees of freedom	14	15	14	14	15	14	14	15	14	14
${\mathrm{R}}^{2}$	0.9723	0.9669	0.9711	0.9703	0.974	0.9735	0.9705	0.9709	0.9686	0.9747
RMSE	0.2155	0.177	0.2129	0.2619	0.1888	0.2002	0.184	0.2075	0.2467	0.1386
AIC	− 41.28	− 52.28	− 41.71	− 34.26	− 49.96	− 43.94	− 46.97	− 46.56	− 36.42	− 57.18

### Relative changes in the regional SUV_mean_

**Fig. 8:**
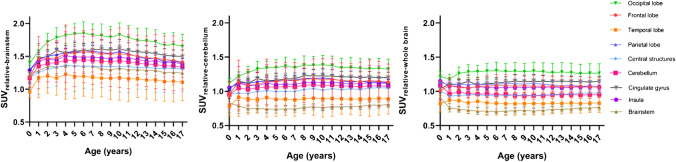
Changes in regional $SU{V}_{mean}$ normalized to brainstem, cerebellum, or whole brain

The relative regional-level changes in the [^18^F]FDG uptake relative to three pseudo-references, whole brain, brainstem, and cerebellum is shown in Fig. 8. Since the occipital lobe had the highest uptake throughout the pediatric range, the $SU{V}_{rel}$ trajectory is above the brain regions. When the SUV is normalized by the brainstem, the parameter trajectories do not have a stable trend but rather increase with age (up to 4 years) and then decrease. On the other hand, the other pseudo-references have a stable behavior and are close to unity (0.5 < $SU{V}_{rel}$<1.5) with age.. The temporal lobe and brainstem showed the lowest values throughout the age range, regardless of the chosen pseudo reference.

### Assessment of laterality

Table 3 shows the structures for which significant differences (*p* < 0.001) in $SU{V}_{mean}$ value were identified between the hemispheres. This effect was found in infants and preschool age (< 6 years old), particularly in temporal areas, frontal structures, and central structures. The caudate nucleus and substantia nigra were the structures that showed significant $SU{V}_{mean}$ differences from the range of 1 to 6 years of age. No significant differences were found in beyond 6 years of age.

**Table 3:** Paired structures that showed asymmetry (*P* < 0.001) in individual left ( −) and right( +) $SU{V}_{mean}$. The percentage difference in $SU{V}_{mean}$ between the two sides is shown in each resulting age group

Age (years)	Structures	$\Delta {\mathrm{SUV}}_{\mathrm{L},\mathrm{R}}$
1	Parahippocampal gyrus	− 7.14
	Temporal superior gyrus	− 13.5
	Lateral orbital gyrus	− 10.51
	Accumbens nucleus	+ 5.19
	Pallidum	+ 8.99
2	Frontal inferior gyrus	− 5.26
	Temporal superior anterior part gyrus	− 22.7
	Lingual gyrus	− 6.33
3	Pallidum	+ 7.34
	Frontal medial gyrus	− 6.46
	Lateral orbital gyrus	− 6.87
	Lingual gyrus	− 8.61
4	Pallidum	+ 9.7
	Accumbens nucleus	+ 10.29
5	Pallidum	+ 7.98
	Accumbens nucleus	+ 8.48
6	Accumbens nucleus	+ 7.45
	Temporal superior anterior part gyrus	− 6.05
	Lingual gyrus	− 5.25
1–6	Caudate nucleus	+ 9.85

## Discussion

This work characterizes brain [^18^F]FDG uptake at different pediatric ages, as a step toward defining reference values for normal uptake (brain atlases) and identification of neurological diseases in children. Our results are consistent with the findings of other authors in terms of demonstrating greater uptake of [^18^F]FDG in the occipital lobe [29, 30, 34] and frontal structures of children’s brains [28, 43]. Previous studies have reported that structures of the occipital lobe (inferior occipital and occipitotemporal gyrus) and cerebellum have a wide range of variability [29, 31]. However, that variability may be due to non-standard uptake practices including patients being awake or in a family member’s presence in the biodistribution room versus in rooms with low light and minimal stimulation during the uptake/biodistribution stage.

In all brain structures, we have shown an increase in [^18^F]FDG uptake until the age of 10, which may reflect synaptic proliferation during this period [44]. The known accelerating loss of gray matter density thereafter may explain the constancy in the ${\mathrm{SUV}}_{\mathrm{mean}}$ at the ages from 13 to 17. According to Fig. 4, there are significant changes up to the age of 13, which could be related to the synaptic pruning and gray matter loss [4]. Results from Hua et al. 2015 [31] show that the monotonous growth of the uptake depends on the brain region and is also restricted until school age (< 12 years old), followed by a fall or stability in the region of adolescence (evaluating by linear spline adjustments). This work had similar results to Shan et al. 2014 [28] with fewer patients (*N* = 115) in younger pediatric age groups. 

Our results show greater dependence of [^18^F]FDG uptake on sex during adolescence (> 13 years old) in accordance with changes due to pubertal development [45]. In most of the brain regions, females had higher uptake of [^18^F]FDG than males. This finding is consistent with [26, 28] previous reports [10, 11, 46–48]. Noteworthy, a trend of high uptake of [^18^F]FDG in the female brain that remains from adulthood to older ages has also been reported [43, 49]. Interestingly, in the present study, significant differences in ${\mathrm{SUV}}_{\mathrm{mean}}$ were noted at adolescent ages, as previously reported [28], and also in infancy. The sex dependence of ${\mathrm{SUV}}_{\mathrm{mean}}$ increased as the subjects grew older, particularly at 7 and 10 years of age, and adolescent ages (13–17 years old). In addition, the parietal lobe had the most significant impact on the significance of sex dependence on ${\mathrm{SUV}}_{\mathrm{mean}}$ at most ages assessed. This is an interesting finding considering that in this brain region, somatosensory, emotional, and language function are carried out [48].

The best fitting model for the temporal changes of SUV in most of the brain regions evaluated was a cubic approximation. These models may be helpful to estimate the normal ${\mathrm{SUV}}_{\mathrm{mean}}$ of [^18^F]FDG at any age and brain region. According to the models, the maximum points of the curves in each region are outside the pediatric age range. This conflicts with prior studies suggesting that these maximums occur between 10 and 15 years of age [26, 28].

Use of pseudo-references to normalize SUV has been evaluated using the cerebellum [28], the whole brain [26, 34], and the brainstem [26, 30]. There is still debate regarding which is the optimal reference given age dependence. When normalizing region uptake with the whole brain, the results are consistent with other authors [26] who have found that the normalized SUV values remain constant along ages with low variability at regional levels. Shan et al. [28] previously showed a quadratic behavior of the SUV values when normalized with the cerebellum. In contrast, our results show a constant trend. For clinical application, the best brain pseudo-reference to normalize the SUV values is likely the cerebellum as there is no sex dependence in cerebellar uptake, and if there is a hypermetabolic area, the ${\mathrm{SUV}}_{\mathrm{rel}}$ with the whole brain would underestimate the ratio and would not be independent of age.

Our results demonstrate asymmetry in [^18^F]FDG uptake at early ages (infancy and preschool) in certain cerebral regions, including frontal (orbital and frontal gyrus), temporal (parahippocampal gyrus as reported previously [29]), occipital (lingual gyrus), and central (nucleus accumbens, pallidum, substantia nigra) structures. These results are consistent with the development of functional brain laterality along with age [47, 50]. Associations with handedness could not be assesses as data related to this were not available for the included sample.

This study is limited by its retrospective design and the inclusion of pediatric patients with extracranial diseases but without CNS involvement as surrogates of healthy children. Effects of this design include a limited sample of patients under the age of 4 and inclusion of patients imaged under anesthesia. While anesthesia decreases glycolytic metabolism, it was administered only for the scan acquisition, not during the uptake/biodistribution phase which is the period that largely determines the patterns of [^18^F]FDG uptake in the brain. In addition, SUV values calculated from whole-body protocol examinations have been assumed to be equal to those calculated based on dedicated brain examinations. This is despite differences in the spatial resolution of these different examinations which may result in different values.

## Conclusions

We have characterized [^18^F]FDG uptake in the brain of a large sample of children without known CNS disease and have generated average maps by age to potentially serve as normal atlases of brain FDG uptake. There is a relatively monotonic increase in ${\mathrm{SUV}}_{\mathrm{mean}}$ in most brain regions from infancy to early adolescence (< 13 years old), followed by stable behavior up to 17 years. Impacts of age, sex, and brain structure on ${\mathrm{SUV}}_{\mathrm{mean}}$ were also observed.

## Data Availability

The datasets generated during and/or analyzed during the current study are freely available from the corresponding author on request.
